# Small intestinal bacterial overgrowth in obese patients with biopsy-confirmed metabolic dysfunction-associated steatotic liver disease: a cross-sectional study

**DOI:** 10.3389/fmed.2024.1376148

**Published:** 2024-05-24

**Authors:** Nicolás Ortiz-López, Ana María Madrid, Larissa Aleman, Alejandra Zazueta, Gladys Smok, Lucía Valenzuela-Pérez, Jaime Poniachik, Caroll J. Beltrán

**Affiliations:** ^1^Faculty of Medicine, Universidad de Chile, Santiago, Chile; ^2^Laboratory of Inmunogatroenterology, Section of Gastroenterology, Department of Medicine, Hospital Clínico Universidad de Chile, Santiago, Chile; ^3^Section of Internal Medicine, Department of Medicine, Hospital Clínico Universidad de Chile, Santiago, Chile; ^4^Section of Gastroenterology, Department of Medicine, Hospital Clínico Universidad de Chile, Santiago, Chile; ^5^Department of Pathologist Anatomy, Hospital Clínico Universidad de Chile, Santiago, Chile; ^6^Division of Gastroenterology and Hepatology, Mayo Clinic, Rochester, MN, United States

**Keywords:** liver diseases, microbiota, non-alcoholic fatty liver disease, small intestinal bacterial overgrowth, liver fibrosis

## Abstract

**Background/aims:**

The metabolic dysfunction-associated steatotic liver disease (MASLD) and obesity are frequent comorbidities with a high prevalence worldwide. Their pathogenesis are multifactorial, including intestinal dysbiosis. The role of small intestinal bacterial overgrowth (SIBO) in MASLD progression in obese patients remains unknown. We aimed to determine the association between SIBO and the severity of MASLD in obese patients.

**Methods:**

An observational and cross-sectional study was conducted in obese patients, diagnosed with or without MASLD by liver biopsy. Metabolic dysfunction-associated steatotic liver (MASL), metabolic dysfunction-associated steatohepatitis without fibrosis (MASH-NF), MASH with fibrosis (MASH-F), or without MASLD (control subjects, CS) were identified by presence of steatosis, portal and lobular inflammation, and fibrosis. SIBO was determined by standardized lactulose breath tests.

**Results:**

A total of 59 patients with MASLD, 16 with MASL, 20 with MASH-NF, 23 with MASH-F, and 14 CS were recruited. Higher percentages of SIBO were observed in MASLD patients (44.2%) compared to CS (14.2%; *p* = 0.0363). Interestingly, MASH-F showed higher percentages of SIBO (65.2%) in comparison to non-fibrotic MASLD (33.3%; *p* = 0.0165). The presence of SIBO was not correlated with the level of hepatic steatosis in MASLD patients.

**Conclusions:**

A positive correlation between MASLD and SIBO in obese patients was principally explained by the presence of liver fibrosis. Our findings suggest a pathogenic role of intestinal dysbiosis in the progression of MASLD. Future research will elucidate the underlying mechanisms of SIBO in MASLD advancement.

## Introduction

Metabolic dysfunction-associated steatotic liver disease (MASLD), formerly known as non-alcoholic fatty liver disease (NAFLD), encompasses patients who have hepatic steatosis and have at least one of five cardiometabolic risk factors, corresponding to an inclusion diagnosis (1). It includes a spectrum of liver conditions, including metabolic dysfunction-associated steatotic liver (MASL) or simple steatosis, and metabolic dysfunction-associated steatohepatitis (MASH) (2). The latter is characterized by inflammation and hepatocyte injury, representing a more severe process of liver damage. MASH is often accompanied by fibrosis and has the potential to progress to cirrhosis (3). The global prevalence of MASLD is estimated to be 30% and has increased 50.4% in the last three decades (4), highlighting its significance as a public health concern. On the other hand, obesity is one of the major health and socioeconomic problems in humans, and it is strongly correlated with MASLD (5). Indeed, the prevalence of MASLD is 50%−90% among individuals with obesity, and the prevalence of obesity in MASLD is 51% (6).

The pathophysiology of MASLD is complex and partially understood (7). Various contributing factors play a role in the development and progression of MASLD, including genetic factors, oxidative stress, impaired adipose tissue function, dysregulation of the immune system, and alterations in the microbiota-gut-liver axis (8, 9). The latter is associated to disruption of the intestinal barrier function provoked by an altered microbiota that leads to an increased gut permeability (10, 11). As a consequence, increased translocation of bacterial products to the liver allows Kupffer cells activation mediated by pattern recognition receptors, such as Toll-like receptors (TLRs), triggering an inflammatory response in the liver (12, 13). Likewise, hepatocytes, hepatic stellate cells, and endothelial cells are also activated, promoting pro-inflammatory and pro-fibrotic reactions and cellular stress by similar mechanism (13–15).

MASLD and obesity are significantly associated with small intestinal bacterial overgrowth (SIBO) (5, 16). SIBO is defined as the presence of abnormal and excessive amount of bacteria in the small intestine (17), which is associated with changes in microbiota composition (18, 19). Several studies have evaluated the role of microbiota modulation through probiotics and synbiotics and reported and improvement in the liver function and metabolic parameters of MASLD patients (20).

The role of SIBO in MASLD progression in obese patients remains unknown, especially regarding its relationship with the fibrotic pathogenic process.

In this study, we aimed to determine the association between SIBO and the severity of MASLD in obese patients.

## Patients and methods

### Study design and setting

We conducted an observational, cross-sectional, prospective, analytic study at the University of Chile Clinical Hospital, a tertiary referral hospital, in 2001. This manuscript follows the STROBE checklist for cross-sectional studies.

### Participants and sampling

The study included adult surgical patients undergoing bariatric surgery. A comprehensive clinical history, including information on nutrition and alcohol consumption, and anthropometric measurements, was collected. Patients with a history of significant alcohol consumption (>30 g/day for males and 20 g/day for females) or liver diseases other than MASLD were excluded. Approximately 2 cm^3^ liver biopsies were obtained during bariatric surgery for histological examination.

### Assessment of MASLD

Liver biopsies were analyzed by a single pathologist in a blinded manner, following previously described methods (21). Briefly, liver samples were fixed in 10% formaldehyde, embedded in paraffin, and stained with either hematoxylin/eosin or Van Gieson's stain. Steatosis was assessed and graded as absent (0), mild (1), moderate (2), or severe (3). The presence or absence of portal and lobular inflammation and fibrosis was also evaluated and graded as absent (0) or present (1). A scoring system was used, with the scores for each parameter being summed to obtain a total score ranging from 1 to 6 points.

The patients were classified into four groups: (1) MASL (or simple steatosis; steatosis in the absence of portal and lobular inflammation and fibrosis), (2) MASH-NF (portal or lobular inflammation in the absence of fibrosis), (3) MASH-F (presence of fibrosis), and (4) control subjects (CS, healthy liver).

### Identification of small intestinal bacterial overgrowth

SIBO was investigated employing the lactulose hydrogen breath test, as previously described (22). Briefly, the test was performed in fasting condition after a mouthwash with 1% hexetidine, and basal values of hydrogen concentration were measured. After the administration of 25 ml of 66.7% lactulose dissolved in 200 ml of distilled water, hydrogen concentration [expressed as parts per million (PPM) in end-expiratory air] was measured using an automatic analyzer (Quintron MicroLyzer Model CM2, Milwaukee, Wisconsin, USA). The following criteria were used to define the presence of SIBO: an increase over basal values of hydrogen concentration of ≥10 PPM during the first 60 min, with an associated second peak caused by the colonic lactulose fermentation. Orocecal transit time expressed in minutes was defined as the time elapsed between lactulose ingestion and the initiation of a sustained increase in hydrogen concentration. In the presence of SIBO, we used the time from the onset of the second hydrogen concentration peak, corresponding to colonic fermentation of lactulose. The patients were classified as with or without SIBO.

### Statistical analysis

The chi-square test was used to assess the difference between categorical variables. The Kruskal–Wallis test was used to compare three or more groups. Statistical significance was assumed at *p* < 0.05. Statistical analyses were performed using GraphPad Prism software version 8.0 for Mac (GraphPad Software, San Diego, CA, USA). The analysis was conducted using R version 4.1.2. A logistic regression model was implemented using the glm() function in R to examine the association between the presence of SIBO and the predictor variables (classification, sex, age, and BMI).

### Ethical considerations

This study was approved by the Ethical Committee of the University of Chile Clinical Hospital, and the study was performed according to Helsinki criteria. All participants signed an informed consent.

## Results

### Characterization of the patients

This cross-sectional study included 73 patients (age range: 19–61 years, mean age: 38.7 years, 84.9% women and 15.0% men), 59 patients with MASLD, including patients with MASL (16), MASH-NF (20), and MASH-F (23), as well as 14 CS with a normal liver. No statistical difference in age, BMI, or sex (*p* = 0.0754, *p* = 0.1087, and *p* = 0.0927, respectively) between groups was found. Table 1 shows the characteristics of the participants.

**Table 1:** Characteristics of the patients.

**Characteristics**	**CS**		**MASL**		**MASH-NF**		**MASH-F**		**Total cohort**		***p*-value**
	***N***	**%**	***N***	**%**	***N***	**%**	***N***	**%**	***N***	**%**	
Total	14	19.1	16	21.9	20	27.3	23	31.5	73	100	
**Sex**											
Male	0	0	2	12.5	4	20	5	21.7	11	15.0	0.2902
Female	14	100	14	87.5	16	80	18	78.2	62	84.9	
Age, mean (SD)	38.5	(10.6)	34.3	(10.4)	37.7	(10.0)	42.9	(10.4)	38.7	(10.6)	0.0754
BMI, mean (SD)	37.9	(4.8)	47.0	(6.0)	44.5	(6.5)	41.8	(11.4)	43.2	(8.5)	0.0927

BMI, body mass index; CS, control subjects; MASL, metabolic dysfunction-associated steatotic liver; MASH, metabolic dysfunction-associated steatohepatitis; MASH-NF, nonfibrotic MASH; MASH-F, fibrotic MASH.

### Histologic analysis

Regarding the hepatic histologic characteristics, all 74 patients were evaluated by biopsy. Fourteen of them did not present liver alterations. Table 2 shows the histological parameters of the 59 MASLD patients.

**Table 2:** Liver histological parameters of MASLD patients.

**Characteristics**	**MASLD patients (*n* = 59)**
**Steatosis grade**	
0: absent	0 (0)
1: mild	16 (27.1)
2: moderate	20 (33.8)
3: severe	33 (55.9)
**Lobular inflammation grade**	
0: absent	22 (37.2)
1: present	37 (62.7)
**Portal inflammation grade**	
0: absent	39 (66.1)
1: present	20 (33.8)
**Fibrosis grade**	
0: absent	36 (61.0)
1: present	23 (38.9)

Data are presented as n and percentage in parenthesis.

MASLD, metabolic dysfunction-associated steatotic liver disease.

### Small intestinal bacterial overgrowth in MASLD patients and controls

A lactulose hydrogen breath test was performed on 14 CS, which was positive in two cases (14.2%). Of the 59 breath tests performed on MASLD patients, 27 were positive (45.7%), and 32 were negative (54.2%). SIBO was significantly higher in MASLD patients compared with the control group (*p* = 0.0363). The degree of steatosis did not correlate with the presence of SIBO in the study participants (*p* = 0.1039; Supplementary Table 1). However, SIBO was positively correlated with fibrosis among MASLD patients (*p* = 0.0026) (Supplementary Table 2).

The SIBO frequency significantly differed between the subgroups of MASLD patients (MASL, MASH-NF, and MASH-F; *p* = 0.0301). Patients with MASH-F had the highest prevalence of SIBO with 65.2%, which was significantly higher compared to subject controls (*p* = 0.0026) and non-fibrotic MASLD (MASL and MASH-NF; *p* = 0.0165). Table 3 shows the frequency of SIBO in MASLD and its subgroups.

**Table 3:** Comparison of SIBO presence between stages of MASLD and control subjects.

**Characteristics**	**CS**		**MASL**		**MASH-NF**		**MASH-F**		**MASLD**		***p*-value**
	***N***	**%**	***N***	**%**	***N***	**%**	***N***	**%**	***N***	**%**	
**SIBO**											
No	12	85.7	9	56.3	15	75	8	34.8^†^	32	54.2^††^	0.0159
Yes	2	14.2	7	43.8	5	25	15	65.2	27	45.7	

^†^p = 0.0026 vs. CS.

^††^p = 0.0305 vs. CS.

BMI, body mass index; CS, control subjects; MASL, metabolic dysfunction-associated steatotic liver; MASLD, metabolic dysfunction-associated steatotic liver disease; MASH, metabolic dysfunction-associated steatohepatitis; MASH-NF, nonfibrotic MASH; MASH-F, fibrotic MASH; SIBO, small intestinal bacterial overgrowth.

To identify potential confounding factors, a logistic regression analysis between SIBO and several variables was made (Table 4). Sex showed a non-significant trend toward significance (estimate = 1.24, *p* = 0.058). Other variables like MASH-F, MASH-NF, MASLD, MASL, age, and BMI did not reach statistical significance. Model fit statistics indicated a good fit (null deviance = 148.79, residual deviance = 130.70, AIC = 148.7).

**Table 4:** Logistic regression analysis for SIBO presence.

**Characteristic**	**OR**	**95% CI**	**p-value**
**Classification**			
CS	–	–	–
MASLD	1.52	0.15, 36.0	0.7
MASL	1.44	0.14,34.5	0.8
MASH-F	2.84	0.21, 78.9	0.5
MASH-NF	0.76	0.06, 20.4	0.8
Sex	3.45	1.03, 14.0	0.058
Age	0.97	0.92, 1.02	0.2
BMI	1.01	0.96, 1.06	0.8

BMI, body mass index; CI, confidence interval; CS, control subjects; MASL, metabolic dysfunction-associated steatotic liver; MASLD, metabolic dysfunction-associated steatotic liver disease; MASH, metabolic dysfunction-associated steatohepatitis; MASH-NF, nonfibrotic MASH; MASH-F, fibrotic MASH; OR, odds ratio; SIBO, small intestinal bacterial overgrowth.

## Discussion

The results of our study demonstrated that obese patients with MASLD had notably higher prevalence rates of SIBO when compared to obese patients without MASLD. This finding is consistent with previous studies that have reported a positive correlation between MASLD and SIBO (16, 23). Additionally, this study found no correlation between the presence of SIBO and the severity of hepatic steatosis in MASLD patients, contrary to the results of Sabaté et al. which observed that the presence of SIBO is associated with the liver steatosis degree in MASLD patients (24). SIBO was positively correlated with the presence of fibrosis (*p* = 0.0026), highlighting the potential role of the SIBO and intestinal microbiota in the progression of MASLD toward liver fibrosis. Indeed, it has been previously reported that patients with liver fibrosis exhibits microbiome characteristics and corresponding alterations in functionality, which could potentially facilitate the development of oxidative stress and a state of inflammation (25). Also, there are studies of association between severity of fibrosis and changes in the intestinal microbiota (26). It is worth mentioning that many observational studies have shown that biopsy-confirmed liver fibrosis is a major predictor of liver-related and overall mortality in MASLD patients (27). Regarding underlying mechanisms, animal studies provides potential links between intestinal dysbiosis and liver fibrosis, including an increased 2-oleoylglycerol macrophage priming (28) unfavorable intrahepatic immune microenvironment, characterized by abnormal distribution and the activation of immune cell subsets due to T cell receptor immune repertoire rearrangement (29); this mechanisms ultimately leads to hepatic stellate cells activation and hepatic fibrogenesis. This evidence suggests that SIBO might contribute to HSC activation and fibrogenesis. Further research is needed to elucidate the underlying mechanisms linking SIBO and MASLD.

On the other hand, it has been suggested that SIBO may contribute to the development of MASLD by inducing gut permeability and systemic inflammation, leading to the development of metabolic disorders, including insulin resistance (30–32). Also, gut microbiota differences between obese patients with or without MASLD has been previously reported by Jin and Xu (33), the gut microbiota composition was similar between obesity with MASLD and simple obesity, but the *Faecalibacterium prausnitzii* colony number was much lower in the obesity with MASLD than in the simple obesity. Modulation of microbiota through probiotics such as Lactobacillus used as a therapeutic approach for MASLD may be beneficial, and has been proved to be beneficial in experimental MASLD (34). Other microbiota modulation approaches may be beneficial, in humans Gravina et al. investigated the effects of bicarbonate–sulfate–calcium–magnesium water, low in sodium, on the microbiota and potential metabolic outcomes in patients with MASLD. Following a six-month intervention, they observed an increase in GLP-1 levels. However, no significant changes were observed in the degree of steatosis, insulin resistance, transaminase levels, or BMI values. Notably, the study reported a significant reduction in the microbiota genus of *Blautia, Collinsella, and Bifidobacterium adolescentis*, typically decreased in MASLD, while an increase was observed in the genus of *Subdoligranulum* and *Dorea*, typically decreased in MASLD patients (35). And furthermore, Gravina et al. (36) also reported a positive impact on functional gastrointestinal symptoms in patients with MASLD.

SIBO is usually treated with antibiotics, such as rifaximin, which also has been shown to reduce endotoxemia levels and liver enzymes in MASLD patients (37). In the study of Gangaparu et al. (38) the treatment of 42 patients diagnosed with MASLD using rifaximin at a dosage of 1,200 mg for 28 days, resulted in a significant reduction in both endotoxemia levels and serum transaminases in the treated patients. While the duration of rifaximin treatment used in the study by Gangarapu et al. differs from the standard duration typically used in SIBO treatment which is generally shorter, usually around 2 weeks, the findings highlight the potential significance of evaluating SIBO treatment in patients with MASLD as a therapeutic approach to ameliorate the disease progression toward fibrosis. However, further studies are necessary to evaluate the role of SIBO treatment in MASLD progression.

It has been proposed that SIBO could promote MASLD through increased inflammation, bile salt deconjugation, decreased intestinal barrier integrity, increased bacterial translocation, and endotoxemia. Likewise, MASLD may promote SIBO through increased oro-cecal transit time, altered bile acid metabolism, and increased insulin resistance. The connection between these two conditions highlights the similarity in the pro-inflammatory signaling pathways associated with both MASLD and SIBO (37).

MASLD in humans is associated with increased gut permeability. This abnormality is related to the increased prevalence of SIBO in these patients (39). In patients with morbid obesity and MASLD, a greater frequency of SIBO is observed with an increasing degree of hepatic steatosis and associated with higher circulating levels of LPS-binding protein (LBP) (40). While our study did not find a correlation between SIBO and the degree of steatosis in MASLD, previous evidence suggests that increased endotoxemia may play a role in SIBO induced progression of MASLD toward fibrosis. A study conducted by Scarpellini et al. (41) investigated the relationship between SIBO, endotoxemia levels, and the severity of liver fibrosis. Their findings demonstrated a significant association between the prevalence of SIBO, elevated levels of endotoxemia, and liver fibrosis. This evidence suggests that SIBO-related endotoxemia may contribute to fibrosis progression in MASLD.

However, it is important to note that the underlying mechanisms linking SIBO, endotoxemia, and fibrosis progression in MASLD are not fully understood and require further investigation. Additional studies are needed to elucidate the complex interactions between gut microbiota, intestinal permeability, endotoxin release, and the development and progression of liver fibrosis in MASLD. In contrast, Guimarães et al. (42) found no significant association between SIBO and elevated serum endotoxin levels in non-cirrhotic patients with MASLD.

It is important to consider that the relationship between SIBO and MASLD is complex and multifactorial. Various factors, such as differences in study populations, diagnostic criteria, and methodologies used, could contribute to the conflicting results observed across different studies.

### Strengths and limitations

This study has several limitations. Firstly, the sample size was small, which may have compromised the statistical power of the study. Secondly, the gold standard for evaluating SIBO is jejunal aspirate culture (with a bacterial colony count ≥10^5^ colony-forming units/ml); nevertheless, other non-invasive tests have been advocated for the diagnosis of SIBO, such as hydrogen breath tests used in this research have gained growing consensus for this purpose (43).

In conclusion, this study provides evidence of an association between SIBO and MASLD in obese patients, particularly in the context of MASLD with fibrosis. These findings contribute to our understanding of the role of the gut microbiota in the progression of MASLD. However, whether this relationship is causal remains unknown, and the role of SIBO in the development of the different stages of MASLD. Further investigations are necessary to explore the causal relationship between SIBO and MASLD and to develop effective therapeutic strategies for these conditions.

## Data availability statement

The original contributions presented in the study are included in the article/Supplementary material, further inquiries can be directed to the corresponding authors.

## Ethics statement

The studies involving humans were approved by Ethical Committee of the University of Chile Clinical Hospital. The studies were conducted in accordance with the local legislation and institutional requirements. The participants provided their written informed consent to participate in this study.

## Author contributions

NO-L: Data curation, Formal analysis, Writing – original draft, Writing – review & editing, Conceptualization, Investigation. AM: Conceptualization, Investigation, Supervision, Formal analysis, Writing – original draft, Writing – review & editing, Data curation, Methodology, Resources, Validation. LA: Data curation, Writing – original draft, Writing – review & editing, Formal analysis. AZ: Writing – review & editing, Methodology, Formal analysis, Software. GS: Investigation, Methodology, Writing – original draft, Writing – review & editing, Formal analysis. LV-P: Conceptualization, Writing – original draft, Writing – review & editing, Formal analysis. JP: Conceptualization, Supervision, Writing – original draft, Writing – review & editing, Investigation. CB: Conceptualization, Supervision, Writing – original draft, Writing – review & editing, Data curation, Formal analysis, Resources, Software.
