# Supplementary material for: Small intestinal bacterial overgrowth in obese patients with biopsy-confirmed metabolic dysfunction-associated steatotic liver disease: a cross-sectional study
Source: Front Med (Lausanne). 2024 May 24;11:1376148. doi: 10.3389/fmed.2024.1376148 (PMC11157043; doi:10.3389/fmed.2024.1376148)
Supplement: Supplementary file 1 [file Data_Sheet_1.docx]

Supplementary Material

# Supplementary Figures and Tables

# Supplementary Table 1. Comparison of SIBO presence between degrees of steatosis among participants (NAFLD patients and control subjects).

|  | **SIBO** | **No SIBO** | ***p*-value** |
| --- | --- | --- | --- |
| **G0 (Absent)** | 2 (6.9%) | 12 (27.2%) | 0.1039 |
| **G1 (Mild)** | 8 (27.5%) | 8 (18.8%) |  |
| **G2 (Moderate)** | 7 (24.1%) | 13 (29.5%) |  |
| **G3 (Severe)** | 12 (41.3%) | 11 (25.0%) |  |

# Abbreviations: NAFLD, non-alcoholic fatty liver disease; SIBO, small intestinal bacterial overgrowth.

#

# Supplementary Table 2. Comparison of SIBO presence between presence/absence of fibrosis among NAFLD patients.

|  | **SIBO** | **No SIBO** | ***p*-value** |
| --- | --- | --- | --- |
| **NASH-F** | 15 (55.5%) | 8 (25.0%) | 0.0026 |
| **NAFL and NASH-NF** | 12 (44.4%) | 24 (75.0%) |  |

# Abbreviations: BMI, body mass index; NAFL, non-alcoholic fatty liver; NASH, non-alcoholic steatohepatitis; NASH-NF, nonfibrotic NASH; NASH-F, fibrotic NASH; SIBO, small intestinal bacterial overgrowth.
